# [Retracted] Dexmedetomidine improves early postoperative neurocognitive disorder in elderly male patients undergoing thoracoscopic lobectomy

**DOI:** 10.3892/etm.2024.12492

**Published:** 2024-03-19

**Authors:** Haixia Shi, Xuejiang Du, Fan Wu, Yajuan Hu, Zhipeng Xv, Weidong Mi

Exp Ther Med 20:3868–3877, 2020; DOI: 10.3892/etm.2020.9113

Following the publication of this paper, it was drawn to the Editor’s attention by a concerned reader that the above paper contained a number of unexpected similarities with an apparently related pre-print article (doi.org/10.21203/rs.3.rs-127973/v1) that appeared in Research Square which, to date, has not been formally submitted elsewhere for publication. The above article and the pre-print article were noted to share the same ethical approval and clinical trial registration numbers, in addition to unexpectedly similar data that had been noted in the Tables.

The Editorial Office conducted an independent investigation of these concerns, and asked the authors to comment on the issues raised by the interested reader. Subsequently, it was noted that, on February 24 2024, Research Square have retracted the preprint article on account of the concerns regarding the alignment of the pre-print article with the above-mentioned article and the divergent research protocols and outcomes, in spite of both studies citing the same clinical trial registration number. Moreover, the authors’ responses to the queries raised were found not to be entirely satisafactory. Therefore, the Editor of *Experimental and Therapeutic Medicine* has decided that this paper should be retracted from the Journal on account of a lack of confidence in the presented data. After having been in contact with the authors, they accepted the decision to retract the paper. The Editor apologizes to the readership for any inconvenience caused.

